# RECRUITMENT AND RETENTION AMONG A LONGITUDINAL COHORT OF AGING ADULTS: EXPERIENCES OF THE WOODLAWN STUDY

**DOI:** 10.1093/geroni/igad104.2350

**Published:** 2023-12-21

**Authors:** Brittany Bugbee, Elaine Doherty, Kerry Green

**Affiliations:** University of Maryland School of Public Health, College Park, Maryland, United States; University of Maryland, College Park, Maryland, United States; University of Maryland at College Park, College Park, Maryland, United States

## Abstract

Minimizing participant attrition is critical to the success of longitudinal studies, but locating and retention is challenging when assessments occur decades apart. This poster will describe efforts to locate participants from the Woodlawn Study, a longitudinal cohort of 1,242 Black Americans followed since 1966 who were assessed at ages 6, 16, 32, and 42. In 2021-2023, two decades since the last assessment, we attempted to locate 1038 participants still thought to be alive to complete an Age 62 interview. Key sources for locating were online contact information databases, genealogical records, and public property records. Deceased participants were identified via National Death Index records. Strategies to encourage participation included monetary incentives, flexible scheduling options, and sharing results from previous assessments. To date, 90.8% have been located, 2.5% are unlocatable, and 6.6% have been identified as newly deceased. Although the cohort originated in Chicago, participants were located in more than 25 states. Interviews were completed with 45.7% of located participants. Approximately one-third (37.7%) did not respond to contact attempts, 6.4% were hard refusals, and 10.3% were soft refusals. Mailed letters and phone calls were the most successful contact methods; social media and email were not fruitful. Interview completion rates were highest for participants who participated at Age 42 (51.4%) and lowest among participants not reached since Age 6 (5.3%). Recruitment challenges included non-response to phone calls, suspicion about scams, and unknown accuracy of locating information. Implications for locating and maintaining aging cohort members will be presented.

